# Cognitive abilities, brain white matter hyperintensity volume, and structural network connectivity in older age

**DOI:** 10.1002/hbm.23857

**Published:** 2017-11-14

**Authors:** Stewart J. Wiseman, Tom Booth, Stuart J. Ritchie, Simon R. Cox, Susana Muñoz Maniega, Maria del C. Valdés Hernández, David Alexander Dickie, Natalie A. Royle, John M. Starr, Ian J. Deary, Joanna M. Wardlaw, Mark E. Bastin

**Affiliations:** ^1^ Centre for Clinical Brain Sciences, University of Edinburgh Edinburgh United Kingdom; ^2^ Department of Psychology University of Edinburgh Edinburgh United Kingdom; ^3^ Centre for Cognitive Ageing and Cognitive Epidemiology, University of Edinburgh Edinburgh United Kingdom; ^4^ Department of Geriatric Medicine NHS Lothian Edinburgh United Kingdom

**Keywords:** neural networks, graph theory, white matter hyperintensities, ageing

## Abstract

**Objective:**

To assess brain structural connectivity in relation to cognitive abilities in healthy ageing, and the mediating effects of white matter hyper‐intensity (WMH) volume.

**Methods:**

MRI data were analysed in 558 members of the Lothian Birth Cohort 1936. Brains were segmented into 85 regions and combined with tractography to generate structural connectomes. WMH volume was quantified. Relationships between whole‐brain connectivity, assessed using graph theory metrics, and four major domains of cognitive ability (visuospatial reasoning, verbal memory, information processing speed and crystallized ability) were investigated, as was the mediating effects of WMH volume on these relationships.

**Results:**

Visuospatial reasoning was associated with network strength, mean shortest path length, and global efficiency. Memory was not associated with any network connectivity metric. Information processing speed and crystallized ability were associated with all network measures. Some relationships were lost when adjusted for mean network FA. WMH volume mediated 11%–15% of the relationships between most network measures and information processing speed, even after adjusting for mean network FA.

**Conclusion:**

Brain structural connectivity relates to visuospatial reasoning, information processing speed and crystallized ability, but not memory, in this relatively healthy age‐homogeneous cohort of 73 year olds. When adjusted for mean FA across the network, most relationships are lost, except with information processing speed suggesting that the underlying topological network structure is related to this cognitive domain. Moreover, the connectome‐processing speed relationship is partly mediated by WMH volume in this cohort. *Hum Brain Mapp 39:622–632, 2018*. © **2017 The Authors Human Brain Mapping Published by Wiley Periodicals, Inc.**

## INTRODUCTION

Cognitive abilities, such as memory and information processing speed, deteriorate with age [Deary et al., 2009] and there are unexplained individual differences in these ageing‐related changes. Disrupted brain white matter communication pathways could be one underlying mechanism for such declines. Measures of age‐related white matter damage such as white matter hyperintensities (WMH) can be derived from magnetic resonance imaging (MRI). WMH relate to cerebral small vessel disease (SVD), increase the risk of stroke [Debette and Markus, 2010] and contribute to dementia [Gorelick et al., 2011]. However, data are lacking on the association between the brain's connectivity patterns and cognitive abilities in generally healthy non‐demented older participants, and on the degree to which WMH might affect the brain's connectomic structure.

Connectomics [Rubinov and Sporns, 2010; Sporns, 2013] uses graph theory [Bullmore et al., 2009] to describe the brain as a network of anatomical links (edges) between brain cortical regions (nodes). Metrics of this topology, which broadly fall into two categories of integration and segregation, include path length and clustering. Shorter path lengths enhance network efficiency, while high clustering coefficients indicate a node's neighbour is also well‐connected to the rest of the network.

Prior work has investigated the mediating influence of connectomic structure on the cognition∼SVD relationship [Reijmer et al., 2016; Tuladhar et al., 2015] in subjects with known SVD. In the current paper, however, we adopt a subtlety different approach by investigating cross‐sectional relationships between four domains of cognitive ability and structural connectivity graph theory metrics derived from brain diffusion MRI in a large group of age‐homogeneous subjects in their early seventies, the Lothian Birth Cohort 1936 (LBC1936). Cohort members are relatively healthy, live in the community, and do not have overt brain disease with clinical symptoms. Such studies are important as these data might provide a better understanding of what is normal for a given age in healthy ageing. Specifically, we investigate the hypothesis that there are links between brain structural connectivity and cognition in the normal ageing brain, and these relationships are mediated by the effects of WMH through the disruption of white matter pathways. Furthermore, we control for mean network FA to allow relationships between network topology (rather than analyses being driven by water diffusion metrics *per se*) and cognitive ability to be investigated.

## METHODS

### Subjects

The LBC1936 comprises 1,091 community‐dwelling individuals who agreed to participate in a longitudinal study of cognitive ageing starting at mean age about 70 years [Deary et al., 2007]. At age 11 years, almost all of them took part in the Scottish Mental Survey of 1947, which employed the Moray House Test No. 12 (MHT) [Scottish Council for Research in Education, 1933], a test of general cognitive ability. At recruitment in older age (at age ∼70 years), between 2004 and 2007, subjects agreed to cognitive testing and other medical, physical and psychosocial assessments. During the second wave of this study, three years after initial recruitment, 738 participants agreed to have a comprehensive MRI examination, including diffusion MRI, to assess brain white matter structure at 73 years of age [Wardlaw et al., 2011]. Inclusion criteria for this study were as follows: no contraindications to MRI, a score ≥24 on the MMSE [Folstein et al., 1975], no diagnosis of neurodegenerative disorders, and complete structural and diffusion MRI, childhood intelligence test scores and cognitive data. No subjects were excluded on the basis of a history of cardiovascular disease, stroke, high cholesterol, hypertension, diabetes or blood circulation problems. Written informed consent was obtained from all subjects. The LBC1936 study was approved by the Multi‐Centre Research Ethics Committee for Scotland (MREC/01/0/56), the Scotland A Research Ethics Committee (07/MRE00/58) and the Lothian Research Ethics Committee (LREC/2003/2/29).

### Cognitive Assessments

In addition to MHT scores of intelligence at age 11, the LBC1936 members completed a battery of cognitive tests at age 73 which were grouped into four domains (on the basis of a previous confirmatory factor analysis [Ritchie et al., 2015]) as follows:

*Visuospatial reasoning* was indicated by the Matrix Reasoning and Block Design subtests from the Wechsler Adult Intelligence Scale, 3rd UK Edition (WAIS‐III‐UK) [Wechsler, 1998a] and the Spatial Span (forwards and backwards) subtest from the Wechsler Memory Scale, 3rd UK Edition (WMS‐III‐UK) [Wechsler, 1998b].

*Verbal memory* was indicated by the Logical Memory (immediate and delayed) and Verbal Paired Associates subtests from the WMS‐III‐UK, and the Digit Span Backward subtest from the WAIS‐III‐UK.

*Information processing speed* was indicated by the Digit‐Symbol Substitution and Symbol Search subtests of the WAIS‐III‐UK and a test of Choice Reaction Time on a dedicated instrument [Deary et al., 2001] and a psychophysical test of Inspection Time [Deary et al., 2004].

*Crystallized ability* was indicated by the National Adult Reading Test (NART) [Nelson and Wilson, 1982], the Wechsler Test of Adult Reading (WTAR) [Wechsler, 2001], and a test of phonemic Verbal Fluency [Lezak, 2004].

In the analyses below, each of the four domains was conceptualized as a latent factor, reflecting each of the relevant cognitive tests.

### Clinical Data

Participants were asked a series of medical questions to determine whether participants had a history of cardiovascular disease or high cholesterol, and whether they were being treated for hypertension, diabetes or blood circulation problems. Measures of blood pressure, cholesterol and HbA1c were available.

### MRI Acquisition

All MRI data were acquired using a GE Signa Horizon HDxt 1.5 T clinical scanner (General Electric, Milwaukee, WI) using a self‐shielding gradient set with maximum gradient strength of 33 mT/m and an 8‐channel phased‐array head coil. Full details of the imaging protocol are available [Wardlaw et al., 2011]. Briefly, subjects provided high‐resolution structural (T_1_‐, T_2_‐, T_2_*‐ and fluid attenuated inversion recovery (FLAIR)‐weighted scans) and diffusion MRI data in the same session. The diffusion MRI examination consisted of 7 T_2_‐weighted (*b* = 0 s mm^−2^) and sets of diffusion‐weighted (*b* = 1000 s mm^−2^) single‐shot spin‐echo echo‐planar (EP) volumes acquired with diffusion gradients applied in 64 non‐collinear directions [Jones et al., 2002]. Volumes were acquired in the axial plane with a field‐of‐view of 256 × 256 mm, contiguous slice locations, and image matrix and slice thickness designed to give 2 mm isotropic voxels. A 3D T_1_‐weighted inversion recovery‐prepared fast spoiled gradient‐echo (FSPGR) volume was also acquired in the coronal plane with 160 contiguous slices and 1.3 mm^3^ voxel dimensions.

### Image Processing

Each 3D T_1_‐weighted FSPGR volume was parcellated into 85 cortical (34 per hemisphere) and sub‐cortical (eight per hemisphere) regions‐of‐interest (ROI), plus the brain stem, using the Desikan‐Killiany atlas and default settings in FreeSurfer v5.3 (http://surfer.nmr.mgh.harvard.edu). The results of the segmentation procedure were visually checked for gross errors and then used to construct grey and white matter masks for use in network construction and to constrain the tractography output. Using tools provided by the FDT package in FSL (http://fsl.fmrib.ox.ac.uk/fsl), the diffusion MRI data were pre‐processed to reduce systematic imaging distortions and bulk subject motion artefacts by affine registration of all subsequent EP volumes to the first T_2_‐weighted EP volume [Jenkinson and Smith, 2001]. Skull stripping and brain extraction were performed on the registered T_2_‐weighted EP volumes and applied to the fractional anisotropy (FA) volume calculated by DTIFIT in each subject [Basser and Pierpaoli, 1996]. The neuroanatomical ROIs determined by FreeSurfer were then aligned from 3D T_1_‐weighted volume to diffusion space using a cross‐modal nonlinear registration method. As a first step, linear registration was used to initialize the alignment of each brain‐extracted FA volume to the corresponding FreeSurfer extracted 3D T_1_‐weighted brain volume using a mutual information cost function and an affine transform with 12 degrees of freedom [Jenkinson and Smith, 2001]. Following this initialization, a non‐linear deformation field based method (FNIRT) was used to refine local alignment [Andersson et al., 2007]. FreeSurfer segmentations and anatomical labels were then aligned to diffusion space using nearest neighbour interpolation.

### Tractography

Whole‐brain probabilistic tractography was performed using FSL's BedpostX/ProbTrackX algorithm [Behrens et al., 2007]. Probability density functions, which describe the uncertainty in the principal directions of diffusion, were computed with a two‐fibre model per voxel [Behrens et al., 2007]. Streamlines were then constructed by sampling from these distributions during tracking using 100 Markov Chain Monte Carlo iterations with a fixed step size of 0.5 mm between successive points. Tracking was initiated from all white matter voxels and streamlines were constructed in two collinear directions until terminated by the following stopping criteria designed to minimize the amount of anatomically implausible streamlines: (i) exceeding a curvature threshold of 70 degrees; (ii) entering a voxel with FA below 0.1; (iii) entering an extra‐cerebral voxel; (iv) exceeding 200 mm in length; and (v) exceeding a distance ratio metric of 10. The distance ratio metric [Bullitt et al., 2003], excludes implausibly tortuous streamlines. For instance, a streamline with a total path length 10 times longer than the distance between end points was considered to be invalid. The values of the curvature, anisotropy and distance ratio metric constraints were set empirically and informed by visual assessment of the resulting streamlines.

### Network Construction

FA‐weighted networks were constructed by recording the mean FA value along streamlines connecting all ROI (network node) pairs. The endpoint of a streamline was considered to be the first grey matter ROI encountered when tracking from the seed location. Self‐connections were removed, and if no streamlines were found between a pair of nodes, the corresponding matrix entry was set to zero. Across the cohort, only connections which occurred in at least two‐thirds of subjects were retained [de Reus and van den Heuvel, 2013]. Finally, for each FA‐weighted connectivity matrix, five global network measures, plus mean edge weight (mean FA for the network), were computed using the brain connectivity toolbox (https://sites.google.com/site/bctnet). The five graph metrics were network density (the fraction of present connections to possible connections), strength (the average sum of weights per node), mean shortest path (the average shortest path length in the network generated from connection‐length connectivity matrices defined as the inverse of the connection‐weight (FA) matrices), global efficiency (the average of the inverse shortest path length) and clustering coefficient (fraction of triangles around a node). Mean shortest path length has an inverse relationship with the other network measures.

### Image Review and Analysis

All MRI scans were reviewed by a consultant neuroradiologist blind to all other data. Imaging features were defined per STRIVE guidelines [Wardlaw et al., 2013]. Intracranial, brain tissue and WMH volumes were measured using Analyze 11.0 (http://analyzedirect.com) and in‐house software “MCMxxxVI” (available from http://sourceforge.net/projects/bric1936/?source=directory) from the structural T_2_‐, T_2_*‐ and FLAIR‐weighted scans. These methods, which were developed locally, have been validated [Valdés Hernández et al., 2015; Wardlaw et al., 2011]. We used quantitative WMH volume (a continuous measure) in our analysis for increased statistical power rather than an ordinal score obtained from a WMH visual rating tool. All segmented volumes were visually inspected for accuracy and to avoid erroneous classification; no subjects were lost to error in the image processing steps. For each subject, WMH volume was normalized by intracranial volume to correct for head size.

### Statistical Analysis

**Figure 1:**
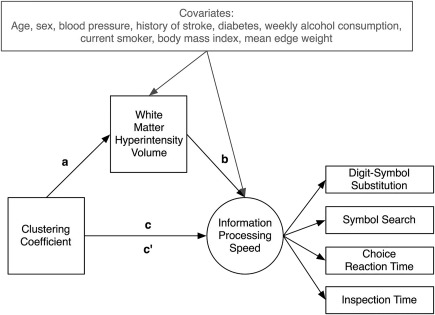
Example of the structural equation modelling.

Structural equation modelling was used to examine the association between network measures and current cognitive domains, and to test whether WMH volume mediated [Hayes and Scharkow, 2013; Iacobucci et al., 2007; Imai et al., 2010] associations between cognition and network connectivity. Mediation exists when a predictor (here, each of the network connectivity metrics) affects the outcome of interest (here, each of four major domains of cognitive ability: visuospatial abilities, memory, processing speed and crystallized abilities) indirectly through at least one intervening variable (here, WMH volume) [Preacher and Hayes, 2004]. A schematic of the models estimated here is shown in Figure 1 using the example of the latent cognitive domain of information processing speed as the outcome and clustering coefficient as the main predictor. Here, as with the other connectome predictor variables (bar density) in the other models, the exemplar of clustering coefficient was residualised by mean edge weight (average network FA) to allow network topological effects to be ascertained free from white matter microstructural integrity metrics. The primary estimates of interest in this study are the degree of change in the direct path between network connectivity measures and cognitive ability, labelled *c* in the bivariate models and *c*′ in the full mediation models, and the indirect path from connectivity measures to cognitive ability through WMH volume: the product of paths a and b. We used the individual cognitive ability test scores as manifest (measured) variables to estimate latent variables within the models for each cognitive domain. Latent variables were identified by fixing one of the factor loadings to unity. Covariates included in the models were age, sex, blood pressure, diabetes, smoking status, history of stroke, body mass index and weekly alcohol consumption. Mean edge weight was also included as a covariate to adjust for any relationship between white matter integrity and the cognitive outcome of interest. Models were estimated using maximum likelihood estimation. Model fit was evaluated based on root mean squared error of approximation (RMSEA), the comparative fit index (CFI) and the Tucker–Lewis index (TLI); good fit was considered as <0.06, >0.90 and >0.90, respectively [Hu and Bentler, 1999]. Bootstrapping (*k* = 1,000 samples) was used to test for the significance of indirect paths [Preacher and Hayes, 2008; Shrout and Bolger, 2002]. Standardised beta coefficients (β) are reported. All analyses were conducted in R v3.3.0 (http://www.r-project.org) (R Core Team, 2013), and the Lavaan library [Rosseel, 2012] was used to conduct the modelling.

## RESULTS

### Subjects

**Figure 2:**
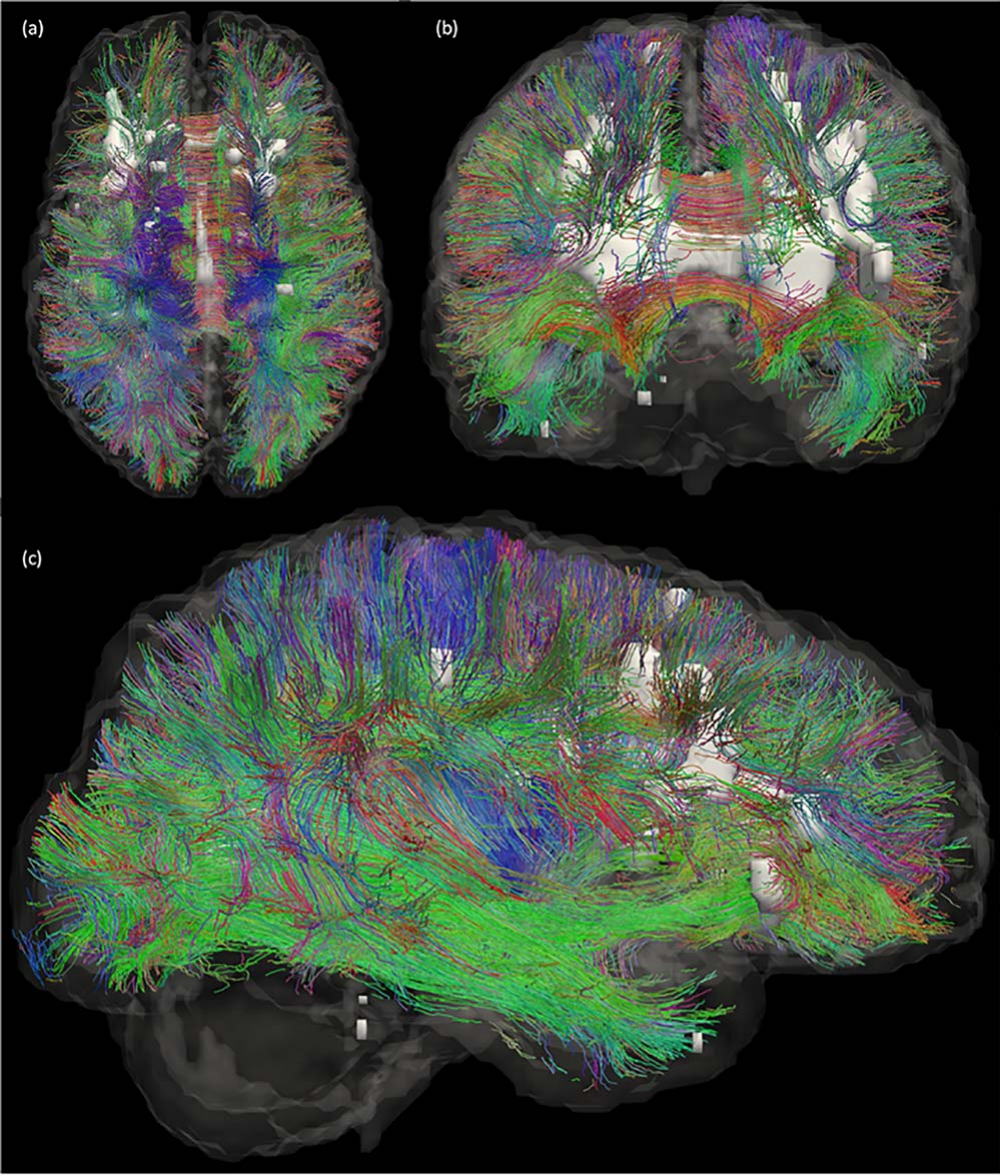
Example of white matter pathways and WMH (white regions) for a representative subject. [Color figure can be viewed at http://wileyonlinelibrary.com]

Five hundred and fifty‐eight subjects met the inclusion criteria and had contemporaneous cognitive data, along with complete processed structural and diffusion MRI. Demographic data for the study group are shown in Table 1. Mean age at MRI scanning was 72.6 (SD 0.68) years. Almost half (48.7%) the cohort were hypertensive, 9.1% reported having diabetes, 7.9% were current smokers and 7% had a history of stroke. White matter pathways and regions of WMH from a representative subject are illustrated in Figure 2.

**Table 1:** Subject characteristics

Demographics	
N	558
Female (%)	259 (46.4%)
Age, years (SD)	72.6 (0.68)
**Vascular risk factors**	
Hypertension (%)	272 (48.7%)
Average systolic blood pressure, mm Hg (SD)	147 (17.8)
Average diastolic blood pressure, mm Hg (SD)	80 (9.4)
Diabetes (%)	51 (9.1%)
Current smokers (%)	44 (7.9%)
Ever smoked (%)	247 (44.3%)
High cholesterol (%)	226 (40.5%)
BMI, kg/m^2^ (SD)	28 (4.4)
History of stroke (%)	39 (7%)
**Cognitive**	
MMSE (SD) [maximum score 30]	29 (1.4)
* Visuospatial reasoning*	
Matrix reasoning (SD)	13.36 (4.85)
Block design (SD)	34.20 (10.1)
Spatial span (SD)	7.38 (1.35)
* Verbal memory*	
Logical memory (SD)	74.47 (18.06)
Verbal paired associates (SD)	27.27 (9.6)
Digit span backward (SD)	7.91 (2.34)
* Information processing speed*	
Digit‐symbol substitution (SD)	56.27 (11.67)
Symbol search (SD)	24.67 (6.02)
Choice reaction time (SD)	0.65 (0.08)
Inspection time (SD)	111.17 (11.48)
* Crystallized ability*	
NART (SD)	34.36 (8.23)
WTAR (SD)	41.1 (7.07)
Verbal fluency (SD)	43.25 (12.76)
**Brain imaging volumes**	
Brain tissue volume, ml (SD)	991.6 (90.0)
Intracranial volume, ml (SD)	1439.5 (133.9)
WMH volume, ml (range)	7.9 (0.4–85.6)
**Network connectivity measures**	
Density (SD)	26.22 (1.25)
Strength (SD)	8.34 (0.72)
Mean shortest path (SD)	4.84 (0.33)
Global efficiency (SD)	0.24 (0.01)
Clustering coefficient (SD)	0.25 (0.02)
Mean edge weight (SD)	0.38 (0.02)

Abbreviations: BMI, body mass index; MMSE, mini mental state examination; NART, National Adult Reading Test; WMH, white matter hyperintensities; WTAR, Wechsler Test of Adult Reading.

Values are mean (standard deviation), median (Q1–Q3 and/or range), or number (%).

### Structural Network Connectivity and Other Variables

The network measures were highly correlated with each other (*β* ± 0.45–0.99; Table 2). Network density (*β* = 0.09; Table 2) but not the other connectivity measures was significantly associated with age. Conversely, network density was not related to WMH volume whereas very strong associations with the other network metrics were noted (all *P* values <0.0001; Table 2). The directionality of network relationships with WMH volume was as expected with higher WMH volumes associated with reduced strength, global efficiency, clustering coefficient and mean edge weight, and increased mean shortest path length. None of the network metrics were related to blood pressure, cholesterol or HbA1c.

**Table 2:** Correlation matrix of connectome measures and simple bivariate relationships between the main predictor variables (connectome and main covariates)

	Density		Strength		Mean shortest path		Global efficiency		Clustering coefficient		Mean edge weight (mean FA across the network)	
	β	*P*	β	*P*	β	*P*	β	*P*	β	*P*	β	*P*
Density	1		0.83	**<0.0001**	−0.64	**<0.0001**	0.61	**<0.0001**	0.61	**<0.0001**	0.45	**<0.0001**
Strength			1		−0.94	**<0.0001**	0.95	**<0.0001**	0.94	**<0.0001**	0.88	**<0.0001**
Mean shortest path					1		−0.99	**<0.0001**	−0.97	**<0.0001**	−0.94	**<0.0001**
Global efficiency							1		0.98	**<0.0001**	0.97	**<0.0001**
Clustering coefficient									1		0.97	**<0.0001**
Mean edge weight											1	
**Main covariates**												
WMH volume	−0.07	0.101	−0.28	**<0.0001**	0.31	**<0.0001**	−0.34	**<0.0001**	−0.30	**<0.0001**	−0.38	**<0.0001**
Age	0.09	**0.037**	0.03	0.542	0.00	0.958	−0.01	0.854	0.00	0.996	−0.03	0.457

FA, fractional anisotropy; WMH, white matter hyperintensities.

Standardised betas (*β*) are reported.

Significant associations (*P* < 0.05) are indicated in bold type.

### Structural Network Connectivity and Cognitive Abilities

Before mediation, simple bivariate analyses showed the following associations between connectome metrics and cognitive outcomes (Table 3). Visuospatial reasoning was associated with network strength, mean shortest path length and global efficiency (*β* ± 0.11 in each case) with a trend toward an association with clustering coefficient. Memory was not associated with any network metric. Information processing speed (*β* range ±0.15–0.22) and crystallized ability (*β* range ±0.10–0.14) were significantly associated with all network measures.

**Table 3:** Structural equation models of network connectivity measures and domains of cognitive abilities mediated by WMH volume (as per example model in Fig. 1)

	Bivariate relationship between cognitive domains and connectome metrics				Full mediation model. Cognitive domain ∼ Connectome residualised by mean edge weight + WMH volume + covariates				
	Total effect c								
	Cognitive domain ∼ Connectome		Cognitive domain ∼ Connectome residualised by mean edge weight		Direct effect c’		Indirect effect ab		
	*β*	*P*	*β*	*P*	*β*	*P*	*β*	*P*	% mediation
**Visuospatial reasoning**									
Density	0.08	0.114	−		—	—	—	—	
Strength	**0.11**	**0.026**	0.03	0.555	—	—	—	—	
Mean shortest path	−**0.11**	**0.022**	−0.03	0.592	—	—	—	—	
Global efficiency	**0.11**	**0.024**	0.02	0.759	—	—	—	—	
Clustering coefficient	0.10	0.054	−0.05	0.345	—	—	—	—	
**Verbal memory**									
Density	0.08	0.102	−		—	—	—	—	
Strength	0.08	0.134	0.06	0.223	—	—	—	—	
Mean shortest path	−0.08	0.105	−0.03	0.434	—	—	—	—	
Global efficiency	0.07	0.176	0.08	0.095	—	—	—	—	
Clustering coefficient	0.06	0.228	0.05	0.324	—	—	—	—	
**Information processing speed**									
Density	**0.22**	**<0.0001**	−		**0.22**	**0.002**	−**0.03**	**0.044**	12%
Strength	**0.21**	**<0.0001**	**0.17**	**<0.0001**	**0.20**	**0.002**	−**0.02**	**0.033**	11%
Mean shortest path	−**0.18**	**<0.0001**	−**0.11**	**0.027**	−**0.15**	**0.011**	**0.03**	**0.034**	15%
Global efficiency	**0.17**	**<0.0001**	**0.10**	**0.013**	**0.17**	**0.006**	−**0.02**	**0.027**	14%
Clustering coefficient	**0.15**	**0.001**	0.02	0.611	—	—	—	—	
**Crystallized ability**									
Density	**0.14**	**0.001**	−		**0.15**	**0.023**	−0.01	0.233	NS
Strength	**0.14**	**0.001**	**0.10**	**0.015**	**0.13**	**0.024**	−0.01	0.229	NS
Mean shortest path	−**0.11**	**0.012**	−0.05	0.285	—	—	—	—	
Global efficiency	**0.11**	**0.007**	0.08	0.051	—	—	—	—	
Clustering coefficient	**0.10**	**0.020**	0.02	0.623	—	—	—	—	

Density not adjusted as FA‐weighted connection weights are ignored in its calculation.

— No direct relationship, and so mediation analysis was not performed.

NS indicates a non‐significant indirect effect.

Significant associations (*P* < 0.05) are indicated in bold type.

WMH = white matter hyperintensities.

Standardised beta coefficients (*β*) are reported with an associated *P* value. The coefficient c captures the relationship between the connectivity and cognitive metrics before mediation (also known as the *total effect*), and is distinguished from *c*′ which captures the *direct effect* of the relationship after controlling for WMH volume. The primary estimates of interest are the degree of change in the direct path between network connectivity measures and cognitive ability, labelled *c* in the bivariate models and *c′* in the full mediation models, and the indirect path from connectivity measures to cognitive ability being the product of paths a and b. Each of the four cognitive domains are latent variables, calculated within the models.

When the bivariate analysis was rerun with the connectome metrics residualised by mean edge weight to control for mean network FA, the visuospatial–connectome relationships were lost. The information processing speed–connectome relationships were maintained (*β* range ±0.10 to 0.17), with the exception of clustering coefficient. The crystallized ability–connectome relationships were lost, except network strength (*β* = 0.10).

### Mediation by WMH Volume

Mediation models were run on the connectome to cognitive ability relationships that showed significant associations in the bivariate analyses. All model fit indices were excellent.

In the domain of information processing speed, controlling for WMH volume and the other covariates did not affect the relationships with the connectome metrics (*β* range ±0.15 to 0.22; Table 3, column *Direct effect c′*). Moreover, the paths through WHM volume for each metric were significant (Table 3, column *Indirect effect ab*), resulting in partial mediations ranging from 11% to 15%.

In the domain of crystallized ability, controlling for WMH volume and the other covariates did not affect the relationship with density (*β* = 0.15) nor strength (*β* = 0.13). However, here the paths through WMH volume were not significant.

## DISCUSSION

Brain network connectivity measures were related to visuospatial reasoning, information processing speed and crystallized cognitive ability, but not memory, in a large sample of community‐dwelling 73‐year olds. The strongest association was between the connectome measures and information processing speed. However, many of the relationships were lost when the connectome measures were investigated free from the effect of mean FA. This suggests that network FA, rather than network topology, is a key driver in most connectome–cognitive ability relationships reported here. The exception to this finding is information processing speed, where the relationship with all network metrics (bar clustering coefficient) withstood adjustment for network FA and other covariates.

The relationship between information processing speed and network metrics was partly mediated by WMH volume where 11%–15% of the relationship was accounted for. Although this degree of mediation is relatively small it remains within the range of that seen in other studies measuring the variance in human cognition accounted for by brain imaging variables [Ritchie et al., 2015]. The limited strength of the mediation might reflect increased water in the interstitial tissues rather than WMH volume as a proxy for advancing demyelination and tract disconnection and dysfunction. Information processing speed was related to the brain network such that poorer levels of segregation (indicated by clustering coefficient) as a marker for subnetwork modularity, and integration (indicated by path length) as a marker for the connectedness of the brain, were associated with worse performance on speed tasks. The seemingly opposing properties of functional segregation within – and anatomical integration across – the human brain [Sporns, 2014] are fundamental to complex, efficient networks. Network efficiency may partly be due to the connectedness of the brain, while WMH appear related to the way the connections interact (rather than a reduction in absolute number of connections *per se*), and is most strongly associated with the ability to process information efficiently.

In prior analyses of the same cohort [Kuznetsova et al., 2016; Penke et al., 2010], we found information processing speed was associated with connectivity across the brain, indexed by tract‐average water molecule diffusion measured using voxel‐based and quantitative tractography methods. Cortical (dis)connection as it pertains to information processing was interpreted as a global process affecting major tracts simultaneously. Whereas earlier work [Penke et al., 2010] used major white matter tracts, this study estimates connectivity between 85 brain regions and provides further evidence of diffuse brain‐wide (or “whole network”) dysfunction independent of mean network FA as an anatomical substrate for reduced processing speed in healthy older age.

The network metrics, except density, were strongly and significantly related to WMH volume such that greater volumes were associated with poorer structural connectivity. Raised blood pressure, cholesterol and diabetes (considered as risk factors related to brain disease) were not associated with network connectivity.

Recently, an association between global network efficiency and cognitive performance in 436 patients (mean age 65.2 years SD 8.8) with clinical SVD was reported [Tuladhar et al., 2015]. A greater volume of WMH, number of lacunes and microbleeds correlated with reduced network density, strength, and global and local efficiency (correlation coefficients ranging from −0.19 to −0.62). Moreover, path analysis showed that network (in)efficiency might drive the association between SVD and cognitive ability. Another study [Lawrence et al., 2014] found that 115 patients of mean age 70.2 years (SD 9.7) with symptomatic SVD had reduced network efficiency versus age‐matched healthy controls, and that global network efficiency related to worse performance on tests of processing speed, executive functioning and gait velocity, but not memory. These studies used hospital‐based populations with wide age ranges. Our cohort members are relatively healthy, living in the community and do not have wide age variation, yet findings appear broadly consistent.

One study [Tuladhar et al., 2015] found SVD severity related to lower density (and network efficiency) in 436 subjects with SVD. Network density is the fraction of present connections to possible connections. It is unclear why density has a weaker and non‐significant relationship to WMH volume in our data, although connection weights are excluded from the calculation of density meaning the topology is represented without “adjustment” for water molecule diffusion anisotropy which broadly represents the integrity of the connections rather than the number of connections *per se*. The other metrics are, in effect, FA‐weighted which could explain the high correlation among these measures.

Study strengths include the use of four measures of cognitive abilities derived from an extensive battery of tests administered by experienced staff, multiple measures of network connectivity (both FA‐weighted and not), a volumetric measure of WMH, a large sample size all scanned on the same research scanner with an identical acquisition protocol and analysis pipeline, and a powerful modelling technique that also adjusted for mean network FA. However, our study is cross‐sectional and while mediation analysis is helpful in using correlational data to test hypotheses about causal pathways, a longitudinal study design where WMH volume at time point *A* predicts poorer connectivity at a later time point *B* would be worthwhile. Longitudinal connectomic data are not currently available in the present study. The direction of the relationships was as expected with, for example, mean shortest path length showing directional relationships opposite to that of the other connectome metrics. We included age as a covariate in these analyses because although this is a birth cohort with all subjects born in 1936, up to a one year difference exists between youngest and oldest and it is important to account for this variation. Finally it should be noted that we investigate the connectome–cognition relationship at a single time point and the influence of accumulated WMH volume on that relationship, notwithstanding the fact that we do not know biologically what precedes what, or indeed if changes are coupled. It is therefore possible that alternative models, for example, one in which WMH is the independent variable and brain connectivity is the mediator, are also relevant.

A limitation of this study is links between brain structure and cognition were assessed at a global level without an investigation of how WMH might affect individual major network connections. One approach to this problem might be to assess structure/function relationships using central networks [Reijmer et al., 2016]. For example, in 72 older patients with mild cognitive impairment, higher levels of WMH were associated with reduced executive function, and this relationship was mediated by FA in central but not non‐central networks [Reijmer et al., 2016]. Further analyses using this methodology may provide a fruitful avenue for future research in this cohort.

Another limitation is that the degree to which our latent measure of processing speed comprises psychomotor speed and other non‐motor speeded cognitive processes is not a question that can be directly interrogated with these data. However, the fact that the Inspection Time test (which explicitly removes any elements of psychomotor speed) loaded on the processing speed factor with comparable magnitude to the other processing speed measures suggests that our latent measure does not principally index psychomotor speed.

As with other connectome studies, a limitation here is that the directionality of brain connectivity cannot be discerned [Sporns, 2013]. Moreover, the spatial scale of tractography and connectomics is several orders of magnitude larger than the underlying architecture of interest, namely axons (MRI voxels are roughly 1 or 2 mm^3^ versus microns for axonal dimensions), such that the network metrics are only estimates of the ‘true’ neural pathways [Toga et al., 2012]. Though we employed a standard atlas and processing pipeline to enable replication and facilitate cross‐study comparison, the number and choice of nodes needs to be considered carefully as this can affect the connectivity output [Zalesky et al., 2010] and there is no universally accepted cortical parcellation scheme [Hagmann et al., 2010]. Finally, our results pertain to healthy older subjects and are not generalisable to a younger general population or to diseased or demented subjects.

In conclusion, we have demonstrated a relationship between global connectome metrics (with and without adjustment for mean network FA) and information processing speed, particularly relative to other cognitive domains, in this large healthy ageing cohort. These relationships were partly mediated by WMH, and although modest, are in the range seen in other studies measuring the variance in human cognition accounted for by brain imaging variables in older age.

## AUTHOR DISCLOSURES

Stewart J. Wiseman: Reports no disclosures

Tom Booth: Reports no disclosures

Stuart J. Ritchie: Reports no disclosures

Susana Muñoz Maniega: Reports no disclosures

Simon R. Cox: Reports no disclosures

Maria del C. Valdés Hernández: Grant funding as listed

David Alexander Dickie: Reports no disclosures

Natalie A. Royle: Reports no disclosures

John M. Starr: Grant funding as listed

Ian J. Deary: Grant funding as listed

Joanna M. Wardlaw: Grant funding as listed

Mark E. Bastin: Grant funding as listed
